# An Innovative Design to Enhance Osteoinductive Efficacy and Biomechanical Behavior of a Titanium Dental Implant

**DOI:** 10.3390/ma17102276

**Published:** 2024-05-11

**Authors:** Yung-Chieh Cho, Pei-Wen Peng, Yu-Sin Ou, Chung-Ming Liu, Bai-Hung Huang, Wen-Chien Lan, Hsin-Hui Kuo, Chia-Chien Hsieh, Brian Chen, Mao-Suan Huang, Hiroyuki Nakano

**Affiliations:** 1 School of Dentistry, College of Oral Medicine, Taipei Medical University, Taipei 110, Taiwan; d204106003@tmu.edu.tw; 2 School of Dental Technology, College of Oral Medicine, Taipei Medical University, Taipei 110, Taiwan; apon@tmu.edu.tw (P.-W.P.); m225098012@tmu.edu.tw (C.-C.H.); 3 General Biology Major with Studio Visual Art Minor, Warren College, University of California, San Diego, CA 92093, USA; y3ou@ucsd.edu; 4 Department of Biomedical Engineering, College of Biomedical Engineering, China Medical University, Taichung 404, Taiwan; liuc@mail.cmu.edu.tw; 5 Graduate Institute of Dental Science, College of Dentistry, China Medical University, Taichung 404, Taiwan; u109312001@cmu.edu.tw; 6 Department of Oral Hygiene Care, Deh Yu College of Nursing and Health, Keelung 203, Taiwan; jameslan@ems.cku.edu.tw; 7 Research Center for Biomedical Devices and Prototyping Production, Taipei Medical University, Taipei 110, Taiwan; d225103001@tmu.edu.tw; 8 Department of Biochemistry, Lehigh University, Bethlehem, PA 18015, USA; bzlanz925966@gmail.com; 9 School of Oral Hygiene, College of Oral Medicine, Taipei Medical University, Taipei 110, Taiwan; 10 Department of Dentistry, Taipei Medical University-Shuang Ho Hospital, New Taipei City 235, Taiwan; 11 Department of Oral and Maxillofacial Surgery, Kanazawa Medical University, Ishikawa 920-0293, Japan

**Keywords:** Ti dental implant, BMP-2, osteoinductive, removal torque, osseointegration

## Abstract

The present study investigated the in vivo bone-forming efficacy of an innovative titanium (Ti) dental implant combined with a collagen sponge containing recombinant human bone morphogenetic protein-2 (BMP-2) in a pig model. Two different concentrations of BMP-2 (20 and 40 µg/mL) were incorporated into collagen sponges and placed at the bottom of Ti dental implants. The investigated implants were inserted into the edentulous ridge at the canine–premolar regions of Lanyu small-ear pigs, which were then euthanized at weeks 1, 2, 4, 8, and 12 post-implantation. Specimens containing the implants and surrounding bone tissue were collected for histological evaluation of their bone-to-implant contact (BIC) ratios and calculation of maximum torques using removal torque measurement. Analytical results showed that the control and BMP-2-loaded implants presented good implant stability and bone healing for all testing durations. After 1 week of healing, the BMP-2-loaded implants with a concentration of 20 µg/mL exhibited the highest BIC ratios, ranging from 58% to 76%, among all groups (*p* = 0.034). Additionally, they also possessed the highest removal torque values (50.1 ± 1.3 N-cm) throughout the 8-week healing period. The BMP-2-loaded implants not only displayed excellent in vivo biocompatibility but also presented superior osteoinductive performance. Therefore, these findings demonstrate that BMP-2 delivered through a collagen sponge can potentially enhance the early-stage osseointegration of Ti dental implants.

## 1. Introduction

Dental implants primarily composed of titanium (Ti) and its alloys have become the gold standard for tooth replacement [1]. The key to their success lies in osseointegration, the process of direct bone-to-implant contact (BIC), critical for implant stability and longevity [2]. This is particularly vital in patients with reduced bone density, significant alveolar bone loss, or metabolic bone disorders, where rapid and robust osseointegration is essential [3,4]. The surface properties of the implant play a crucial role in this context. Various modification techniques, including physicochemical, morphological, and biochemical methods, have been developed to improve the bone–implant interface [5,6,7,8].

Bioactive coatings, achieved via diverse surface modification techniques such as plasma electrolytic oxidation in aqueous electrolytes and molten salts, have been extensively explored [9]. Furthermore, recent advances in dental implant technology have harnessed bioactive molecules, especially growth factors, to enhance osteoconduction and osteoinduction, thereby facilitating better integration with the surrounding bone [10,11,12]. Bone morphogenetic protein-2 (BMP-2), a member of the transforming growth factor-beta (TGF-β) superfamily, is notable for its powerful osteoinductive capabilities [13]. BMP-2 plays a pivotal role in promoting osteogenesis and angiogenesis and is increasingly being incorporated into bone graft materials to bolster their effectiveness [14,15,16,17,18]. However, the clinical application of BMP-2 faces challenges, including unpredictable release, a short biological half-life, and rapid in vivo diffusion [19,20,21]. Consequently, the development of a biomaterial scaffold capable of effective BMP-2 delivery is critical for its sustained release.

Collagen, a crucial component of the extracellular matrix in bone tissue, has been used to coat Ti implants. These collagen-coated Ti implants have been shown to promote tissue vascularization, inhibit the inflammatory response, and reduce osteoclast activity. However, the expected stimulatory effect on accelerated osteoblast differentiation was not observed with the collagen-coated Ti implants [4,22]. Combining collagen with BMP-2 should be a straightforward approach to address the lack of accelerated osteoblast differentiation. Collagen is a potential candidate for BMP-2 delivery, making it an effective medium for enhancing this aspect of bone regeneration [23]. Its ability to form sponge-like structures while maintaining matrix integrity makes it an ideal candidate for bone tissue regeneration [24]. Studies, such as those by Bouxsein et al. [25], have demonstrated that the use of a collagen sponge matrix for recombinant human BMP-2 (rhBMP-2) delivery creates a slow-release system with greater protein retention compared to buffer delivery. An et al. [26] explored computer-guided flapless crestal sinus augmentation surgery using BMP-2-loaded Bio-Oss collagen for immediate nonfunctional loading of dental implants, showing promising results in terms of bone density and implant stability.

Despite the recognized potential of BMP-2 in bone regeneration, research focusing on the specific effects of BMP-2 delivered via collagen carriers on bone formation and implant stability during the initial stages of treatment remains limited. Furthermore, it is imperative to define the optimal dosage of BMP-2, as excessive amounts could activate BMP-2 inhibitors, potentially undermining therapeutic efficacy. Consequently, this study aimed to assess the effect of various concentrations of rhBMP-2 delivered through collagen on osseointegration by evaluating new bone formation and implant stability using an innovative Ti dental implant in a Lanyu small-ear pig model. The process of osseointegration will be examined at multiple time points: 1, 2, 4, 8, and 12 weeks post-implantation. The data gathered will provide valuable scientific insights for the further development and refinement of Ti dental implants.

## 2. Materials and Methods

### 2.1. Implants Preparation

Dental implants used in the present study were made from biomedical grade 4 pure Ti bars with a diameter of 4 mm and a length of 10 mm. The machined implants were further surface treated by an SLA technique (sand-blasted, large grit, and acid-etched) from 3D Global Biotech Inc., New Taipei City, Taiwan and Sentronic International Corp., New Taipei City, Taiwan. Thereafter, a hole with a diameter of 2 mm and a length of 2 mm was created at the bottom of the implants using a precision computer numerical control machine (CT-100, CHC Machinery Co., Ltd., Taichung, Taiwan). This innovative design was utilized for delivering BMP-2, which was encapsulated in a collagen carrier and inserted into the prepared hole. Before implantation, the surface morphology and chemical composition of the investigated Ti implant were identified by a JEOL JSM-6500F field emission scanning electron microscope (FE-SEM, Tokyo, Japan) equipped with an energy-dispersive X-ray spectroscope (EDS; INCA, Oxford Instruments, Abingdon, UK).

### 2.2. Experimental Groups

Four experimental groups were investigated in the present study, as outlined in Table 1. The control group consisted of implants without collagen sponges. For the BMP2-0 groups, bovine-derived collagen sponges (AteloCell, KOKEN Co., Ltd., Tokyo, Japan) of 2 mg were cut to specific dimensions, soaked in an albumin-containing solution, and then inserted into the implant hole at the bottom. For the BMP2-20 and BMP2-40 groups, rhBMP-2 derived from Chinese hamster ovary (CHO) cells (United Biomedical Inc., Taipei, Taiwan) were diluted in a buffer solution to 20 and 40 ng/µL concentrations, respectively. Subsequently, the collagen sponge was then immersed in 100 µL of the prepared rhBMP-2 solution, resulting in a loading of 20 and 40 µg of rhBMP-2 for the BMP2-20 and BMP2-40 groups, respectively.

### 2.3. Experimental Animal Model

**Figure 1:**
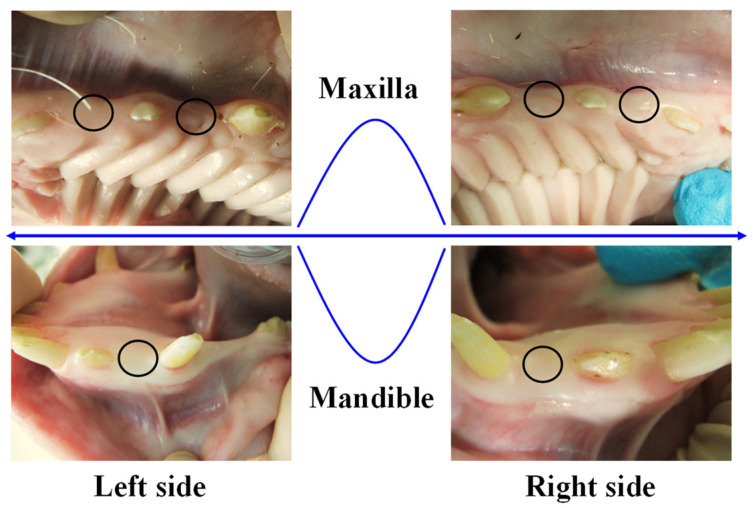
The implantation locations (marked as black circular areas) of the investigated Ti implants in the oral area of the pigs.

The present study obtained approval from the Institutional Animal Care and Use Committee of Taipei Medical University (LAC-100-0024). A total of 20 Lanyu small-ear pigs with an average weight of 57.95 ± 3.16 kg and an average age of 12 months were utilized for the study. A systematic random protocol was employed to determine the implant locations, ensuring a minimum distance of 15 mm between each implant to avoid interfering with the healing process. Before implantation, the animals received intramuscular injections of Stresnil (3–4 mg/kg) and Atropine (0.04–0.05 mg/kg), and anesthesia was maintained using Isoflurane (1.5–2%) and a mixture of nitrous oxide/oxygen (1:2). Afterward, a total of 8 implants were inserted into the edentulous ridge of each animal, two on each side of the maxilla and mandible, specifically in the canine-premolar regions (Figure 1). The implant sites were created with a 3 cm-long incision and prepared using a guide drill, followed by a sequence of twist drills. The implants were inserted into a bone-level position using a precise adjustable implant torque wrench under a torque of 35 N-cm (Hung Chun BIO-S Co., Ltd., Kaohsiung, Taiwan). The flap was then closed using the submerged technique. Postoperatively, each pig was administered antibiotics (diclofenac potassium 100 mg) and analgesics (cephalexin 1000 mg), which were mixed into their food and given for seven consecutive days. The DIOX-602 portable dental X-ray system (DIGIMED Co., Ltd., Seoul, Republic of Korea) was adopted to observe and evaluate the defect, failure, and inflammation in the investigated implants post-implantation. Animals designated for sacrifice at 1, 2, 4, 8, and 12 weeks (*n* = 4, per week) were euthanized under general anesthesia by intravenous injection of an overdose of sodium pentothal. Implants and surrounding tissues of at least 0.5 cm in all directions were harvested using a diamond blade and fixed in 10% neutral buffered formalin. Half of the specimens were used for histomorphometric analysis, while the remaining specimens were allocated for the removal torque testing.

### 2.4. Evaluation of Bone Tissue Regeneration

**Figure 2:**
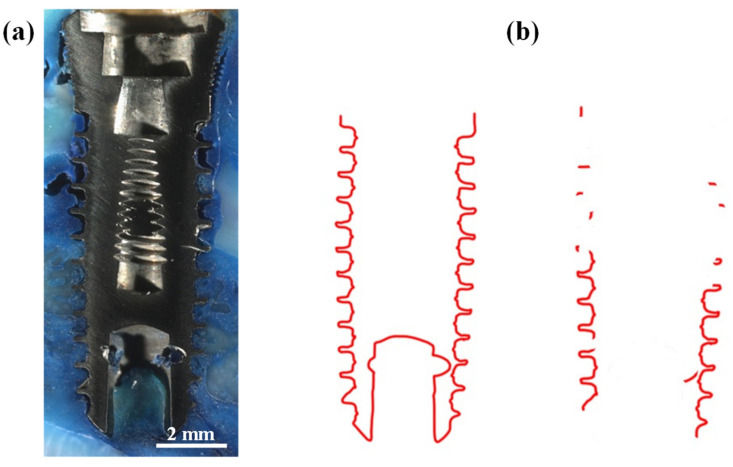
(**a**) Histomorphometric evaluation of BIC using undecalcified ground sections and (**b**) depiction of the length of the implant’s surface where direct BIC occurred without any intervening tissues.

Histomorphometric evaluation of BIC was conducted using undecalcified ground sections. The harvested tissues were dehydrated using a series of graded alcohol concentrations (70%, 80%, 90%, and 100%). Subsequently, the tissues were embedded in epoxy resin and then sectioned parallel to their longitudinal axis using a low-speed diamond saw (model CL40, Fortune Tell Co., Ltd., New Taipei City, Taiwan) under 150 g loading, 200 rpm, and 250 mL deionized water. Then, the sections were ground down to a thickness of 100 μm and further polished to a final thickness of 50 μm. The polished specimens were surface stained with toluidine blue and observed using a light microscope (Discovery V20, ZEISS, Buffalo Grove, IL, USA). The evaluation involved depicting the length of the implant’s surface where direct BIC occurred without any intervening tissues. The BIC ratio and the area of new bone formation within the thread area were determined in each section using image analysis software (Version 6.0, Image-Pro Plus, Media Cybernetics, Rockville, MD, USA) (Figure 2). The BIC ratio is defined as the percentage of the implant length where direct BIC occurred without intervening tissues.

### 2.5. Removal Torque Measurement

To assess the strength and rate of osseointegration, this study employed a micro-computer screw torsion tester (model CY-6040A10, Chun Yen Testing Machines Co., Ltd., Taichung City, Taiwan) to measure the force required to unscrew the implants with a counterclockwise rotation rate of 1 radius/min. The maximum removal torque (RTQ, N-cm) was recorded as a result.

### 2.6. Statistical Analysis

Statistical analysis was performed using IBM SPSS Statistics (v19.0, IBM Corp., Armonk, NY, USA), and the results were presented as mean ± standard deviation. The Mann–Whitney test assessed differences in BIC ratio and RTQ values among the experimental groups.

## 3. Results

### 3.1. Implant Features

**Figure 3:**
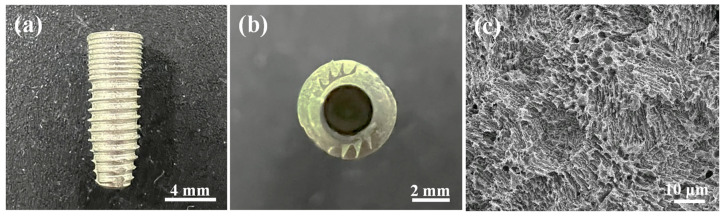
The morphology images of the investigated Ti implant: (**a**) SLA-treated implant and (**b**) the created hole at the bottom of the implant, and (**c**) a higher magnification FE-SEM micrograph taken from the SLA-treated surface in (**a**).

Figure 3a shows the morphology features of the investigated Ti implant after SLA treatment. In addition, a hole structure with carrier function was created at the bottom of the implant (Figure 3b). The diameter of the hole size at the bottom of the implant was approximately 2.0 ± 0.05 mm. From a higher magnification micrograph observation, it is clearly seen that a typical micro-porous structure feature was formed on the surface of the SLA-treated Ti implant (Figure 3c). Moreover, the EDS analysis results indicated that the elemental compositions of the SLA-treated surface are composed of Ti, Si, O, C, and N elements (Table 2). No other contaminants or impurity substances were formed on the SLA-treated Ti surface before implantation.

### 3.2. Radiograph Observation

**Figure 4:**
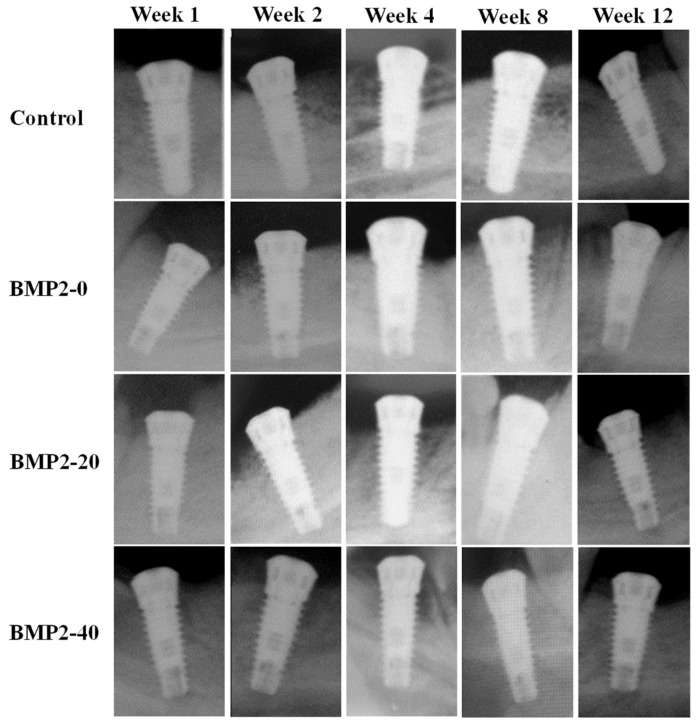
Radiographic images from the investigated implants after implantation for different durations.

Figure 4 presents the radiographic images from the investigated implants after different testing durations of implantation. Apparently, the control and BMP-2-loaded implants displayed well-stable implantation, as shown in all testing durations. In addition, the cortical and cancellous bone tissues not only exhibited good health statuses but also showed favorable bone healing around the investigated implants. There were no other defects or inflammation that could be observed in the investigated implants after being implanted for 1, 2, 4, 8, or 12 weeks. It is evident that excellent in vivo biocompatibility was found for the BMP-2-loaded implants.

### 3.3. Histological Analysis

**Figure 5:**
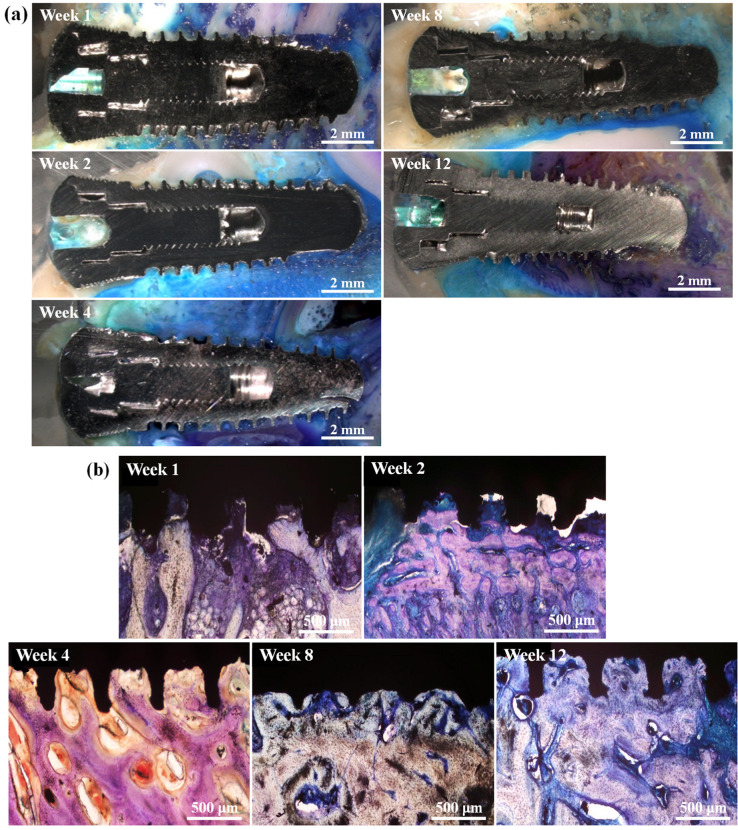
Histology analysis results of the control group after implantation for different durations: (**a**) representative histological section images and (**b**) higher-magnification images taken from the implant/bone tissue interface in (**a**).

**Figure 6:**
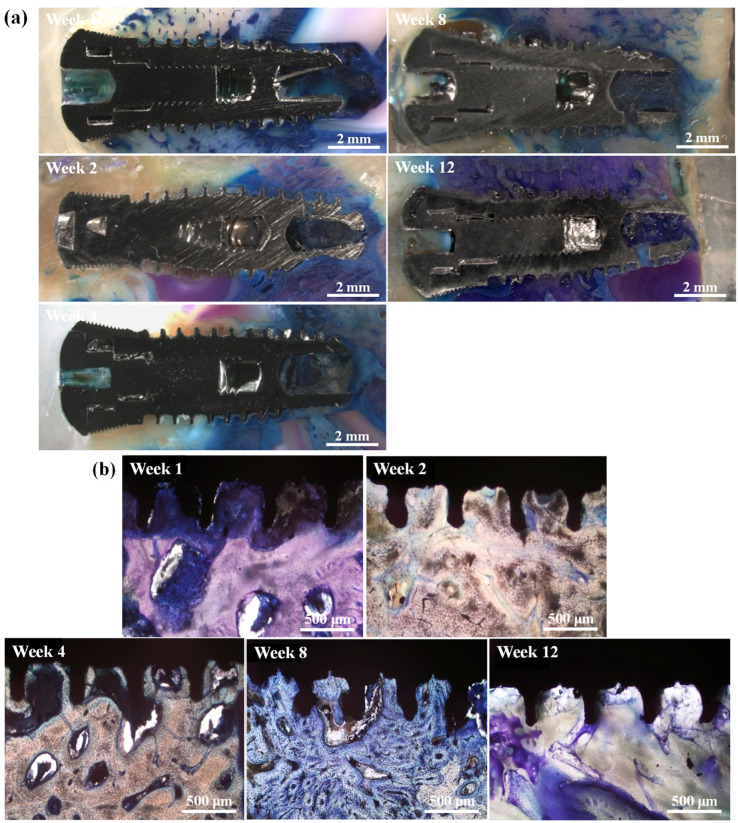
Histology analysis results of the BMP2-20 group after implantation for different durations: (**a**) representative histological section images and (**b**) higher-magnification images taken from the implant/bone tissue interface in (**a**).

A total of 148 implants were examined for histomorphometric evaluation and biomechanical analysis (Table 3). For the histomorphometric evaluation, two implants were excluded from the study due to one implant loss and another failure during the preparation process. Regarding the biomechanical analysis, ten implants were excluded from the study due to dehiscence (a separation or opening) in the cover screws. Importantly, these exclusions were made despite the absence of any signs of inflammation in those cases. Figure 5 illustrates the histomorphometric analysis of the control group, while Figure 6 depicts the histomorphometric analysis of the BMP2-20 group. Overall, no indications of cellular inflammatory infiltrations, foreign body reactions, or distinctive macrophage reactions were detected in the histological slides. During the first week of healing, direct contact between the implants’ surfaces and the surrounding bone tissue was observed, indicating initial stability for all implants. No osteolysis was found after 2 weeks of healing, and well-organized osteoid formation was evident. By week 4, woven new bone was observed within the implants’ threads, along with the early stages of haversian systems’ formation. After 8 weeks of healing, the newly formed bone around the implants exhibited a broad-based configuration. Similar observations persisted post-implantation for 12 weeks.

### 3.4. New Bone Formation and Biomechanical Responses

**Figure 7:**
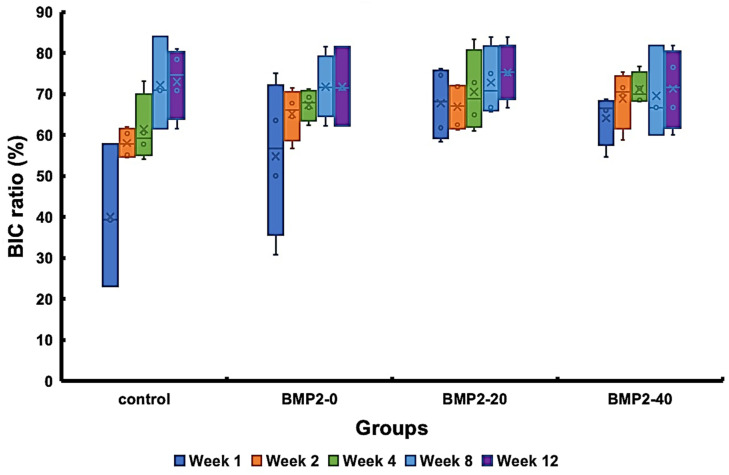
Histomorphometric evaluation of the BIC ratios of the investigated implants post-implantation at different durations.

**Figure 8:**
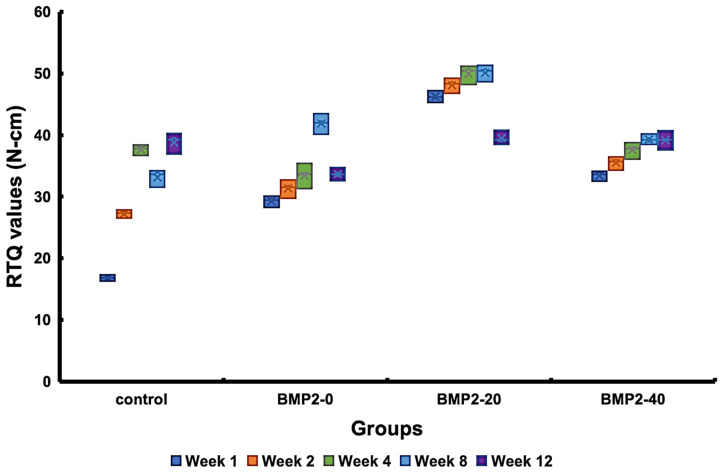
The RTQ results of the investigated implants post-implantation at different durations.

Figure 7 presents the results of the histomorphometric evaluation in terms of the BIC ratio, demonstrating a gradual increase in BIC ratios over time for all groups. The BMP2-20 group possessed the highest BIC ratios among the groups, followed by the BMP2-40, BMP2-0, and control groups, in descending order, after 1 week of healing (*p* = 0.034). Subsequently, the BMP2-20 group maintained slightly greater BIC ratios than the other groups, although these differences did not reach statistical significance. Figure 8 displays the RTQ values of the implants with different concentrations of BMP-2. The results showed that at 8 weeks of healing, the BMP2-20 group demonstrated the highest RTQ value of 50.1 ± 1.3 N-cm, followed by the BMP2-40, BMP2-0, and control groups, in descending order. However, after 12 weeks of healing, all groups exhibited similar RTQ values without significant differences.

## 4. Discussion

The present study histologically and biomechanically evaluated titanium implant surfaces modified with rhBMP-2 delivered via a collagen sponge. The osteoinductive efficacy and osseointegration potential of two dosages of rhBMP-2 were assessed. Animal models, particularly Lanyu small-ear pigs, play a pivotal role in dental implant research due to their bone regeneration rates, which closely resemble those of humans (1.2–1.5 μm per day) [21]. These pigs offer a valuable tool for investigating bone regeneration and implant osseointegration. However, it should be noted that during implant retrieval, a high incidence of dehiscence was observed in the pigs, attributed to the parafunctional masticatory load, similar to a previous study [27]. Furthermore, the study revealed significant variations in BIC ratios and resonance frequency analysis (RTQ) values within the group of pigs, indicating that each pig exhibited a unique bone formation potential. Unfortunately, the extensive data variations did not yield statistically significant results among the groups, which may require further investigation. Non-parametric tests like the Mann–Whitney test were used in the present study. The sample size for each group in the present study was less than 30. Given this relatively small sample size, the power of parametric tests, which assume normal distribution, might be compromised.

rhBMP-2 has demonstrated promising osteogenic potential; however, its clinical utility is limited due to rapid proteinase degradation in the body [21]. Consequently, achieving sufficient bone formation requires high doses of rhBMP-2, which can escalate costs and lead to unintended complications, such as heterotopic bone formation, soft-tissue swelling, and even malignancy [10,21]. An alternative approach involves utilizing a suitable carrier to enhance the effective use of rhBMP-2 and enable sustained release over time [20]. This approach aims to optimize the therapeutic outcomes while mitigating the need for excessively high rhBMP-2 doses and reducing associated adverse effects.

As a BMP-2 carrier used in the present study (the BMP2-0 group), collagen also exhibited a higher BIC ratio and RTQ value than the control group. Lutz et al. [21] investigated the effect of topical BMP-2 gene delivery in conjunction with a collagen carrier on bone formation and implant osseointegration. Comparing the results to the group treated with collagen alone, higher mineralization values and improved BIC in the BMP-2/collagen group using a domestic pig model were consistent with the present study, even though statistical significance was not achieved.

Determining the appropriate threshold or effective doses of BMP-2 concentrations is crucial for bone formation. Mumcuoglu et al. [28] conducted a study using two different concentrations of BMP-2, 5 μg/mL and 50 μg/mL, in a subcritical calvarial defect model. They employed an injectable hydrogel consisting of BMP-2-loaded recombinant collagen-based microspheres and alginate. Complete defect bridging was observed after 8 weeks when the 50 μg/mL concentration was used. However, BMP-2-loaded implants with high concentrations may recruit and activate more bone-resorbing cells than osteoprogenitor ones, eventually inducing opposing effects such as noggins and ankylosis [29,30]. A higher BMP-2 concentration in the present study did not necessarily lead to greater bone formation. These variations in results could be attributed to differences in delivery systems, animal models, and experimental conditions. Interestingly, the BMP2-20 group demonstrated the highest BIC ratios and RTQ values among the groups, especially at early time points, suggesting that 20 μg per implant was sufficient to promote early bone healing. This highlights the significance of selecting an appropriate BMP-2 dosage to achieve optimal bone healing outcomes.

Bone formation directly on the implants’ surfaces was observed in both the control and BMP2-20 groups, as evidenced by histological examination. The BMP2-20 group exhibited higher BIC values during the first- and second-week follow-ups, suggesting that rhBMP-2 enhances the osteoconductivity of the implants’ surfaces. However, no significant differences in BIC values were observed among the groups after this period, implying that the effect of rhBMP-2 adsorbed into collagen may not persist beyond 4 weeks. This observation warrants further investigation.

The most critical factor for successful implantation is primary implant stability, which is associated with insertion torque [31]. High implant micromobility has been linked to poor bone quality and quantity, as well as low insertion torque values [32]. In this study, an insertion torque of 35 N-cm was selected based on the bone quality of the animal model and served as the baseline for subsequent removal torque measurements. In this study, higher BIC values correlated with higher RTQ values throughout the healing period, suggesting that rhBMP-2-modified implants provided the necessary initial stability for effective osseointegration. This observation supports considering these implants as a potential alternative to traditional delayed loading protocols. Traditionally, the surgical area requires 3 to 6 months of healing for two-stage procedures. However, some clinicians have reduced this period to as little as 6 weeks, or even omitted it entirely, under conditions of adequate bone quality and quantity, along with sufficient primary stability [33]. The higher BIC and RTQ values observed in the BMP-2-loaded Ti implants at the early stages provide initial evidence supporting the feasibility of immediate loading. Further research is, however, essential to conclusively demonstrate and validate this capability across broader clinical scenarios.

Although the results confirmed that the BMP2-20 group appeared to promote new bone regeneration within 12 weeks, a longer testing duration is needed to offer further evidence to observe the osseointegration phenomenon. Finally, further works on biomechanical stress distribution simulation, optimal BMP-2 concentration, and the release rate and mechanism of BMP-2 in vivo must be conducted to fully reflect the influence of BMP-2 loading on the osteoinductive efficacy and osseointegration potential of the BMP2-loaded Ti implant.

## 5. Conclusions

An innovative Ti dental implant combined with a collagen sponge containing rhBMP-2 was successfully fabricated. The BMP-2-loaded Ti implants demonstrated superior in vivo biocompatibility across various post-implantation durations. In the early stages of healing, these implants positively influenced bone formation and removal torque values. However, no statistically significant differences were observed in the pig model. Despite these findings, this study suggests that the combination of BMP-2 with a collagen carrier has the potential to serve as an osteoinductive activator for dental implants.

## Figures and Tables

**Table 1:** Experimental groups used to evaluate osteoinductive efficacy in the pig model.

Group	rhBMP-2 Concentration	Description
Control	0	Without collagen sponge and BMP-2
BMP2-0	0	Without BMP-2
BMP2-20	20 μg/collagen sponge	20 μg of BMP-2 infiltrating into the collagen sponge
BMP2-40	40 μg/collagen sponge	40 μg of BMP-2 infiltrating into the collagen sponge

**Table 2:** The surface chemical composition of the SLA-treated Ti implant (wt.%).

Ti	Si	O	C	N
Bal.	1.23 ± 0.12	0.83 ± 0.23	0.21 ± 0.1	<0.01

**Table 3:** The investigated implant failures during the healing durations.

Group	Histomorphometric Evaluation (*n*)	Biomechanical Analysis (*n*)	%
Control	1	2	7.5
BMP2-0	0	4	10
BMP2-20	1	1	5
BMP2-40	0	3	7.5
Total	2	10	7.5

## Data Availability

Data are contained within the article.
